# First detection and molecular characterisation of pseudocowpox virus in a cattle herd in Zambia

**DOI:** 10.1186/s12985-020-01426-7

**Published:** 2020-10-09

**Authors:** Maureen Wakwamba Ziba, Chanda Chitala, Tirumala Bharani K. Settypalli, Malama Mumba, Giovanni Cattoli, Paul Fandamu, Charles Euloge Lamien

**Affiliations:** 1 Department of Veterinary Services Ministry of Fisheries and Livestock, Central Veterinary Research Institute, P.O Box 33980, Lusaka, Zambia; 2 Department of Nuclear Sciences and Applications, Joint FAO/IAEA Division of Nuclear Techniques in Food and Agriculture, International Atomic Energy Agency, Wagramer Strasse 5, P.O. Box 100, 1400 Vienna, Austria

**Keywords:** Pseudocowpox virus, HRM assay, B2L gene, Parapoxvirus, Zambia

## Abstract

**Background:**

Pseudocowpox virus (PCPV) of the genus *Parapoxvirus* in the family *Poxviridae* causes pseudocowpox in cattle worldwide and presents a zoonotic concern. Most poxviruses produce diseases of similar clinical signs in affected animals, which are impossible to differentiate clinically or by serology. It is, therefore, vital to use molecular assays to rapidly identify the causative agents of poxvirus infections. This study aimed to detect, diagnose, and characterize the causative agent of pox-like skin lesions in a cattle herd in Zambia, initially suspected to be infected with Lumpy Skin Disease virus.

**Methods:**

We used a High-Resolution Melting (HRM) analysis assay to detect the PCPV genome and sequenced the major envelope protein (B2L gene) for comparative sequence and phylogenetic analysis.

**Results:**

Our field investigations showed cattle presenting atypical skin lesions and high morbidity within the herd. The laboratory diagnosis, based on the HRM assay revealed PCPV DNA in the samples. Phylogenetic and comparative sequence analyses confirmed PCPV in the samples and revealed genomic differences between samples collected in 2017 and 2018 from the same farm.

**Conclusion:**

Our work is the first documented report of PCPV in Zambia. It shows the strength of molecular methods to diagnose pox-like infections in cattle and discriminate between diseases causing similar clinical signs. This rapid and accurate diagnosis improves the response time for more accurate veterinary interventions.

## Background

Pseudocowpox is a pox-like disease of cattle caused by pseudocowpox virus (PCPV) of the genus *Parapoxvirus* (PPV) within the family *Poxviridae* [1]. This genus also includes bovine papular stomatitis virus (BPSV) of cattle and orf virus (ORFV) of sheep and goats. Additional PPVs affect red deer of New Zealand (PVNZ), reindeers, seals, and musk ox [2–5]. Other poxviruses, within the genera *Capripoxvirus* and *Orthopoxvirus,* can also affect cattle.

Parapoxviruses cause papules and erosions on the muzzle, oral mucosa, and udder [6] and may cause high morbidity and loss of productivity [7]. Parapoxvirus infections may also be asymptomatic [8], and can infect humans working in close contact with infected animals [7, 9].

Parapoxvirus infections can be clinically diagnosed, however, clinical signs may overlap with other diseases such as lumpy skin disease (LSD), bovine herpes virus (BoHV), BPSV, and orthopoxvirus. In Zambia, LSD has been documented since 1929, but information on PPVs and orthopoxvirus infections of cattle are lacking. Typically, cases presenting with pox-like lesions are treated as LSD, BoHV, or other skin diseases of cattle. Samples are rarely sent to the laboratory for further diagnosis due to a lack of differential diagnostic tools. Recent advancements have produced molecular assays that are fast, sensitive, and offer a means to discriminate parapoxviruses from other agents producing similar cutaneous lesions [10–12].

For the first time, we report the diagnosis of pseudocowpox in Zambia from LSD suspected cases. This paper describes the clinical presentation, molecular detection, and molecular characterization of the index cases of PCPV from clinical samples and discusses the subsequent field investigation in a herd of cattle.

## Methods

### Clinical and epidemiological investigations

**Fig. 1:**
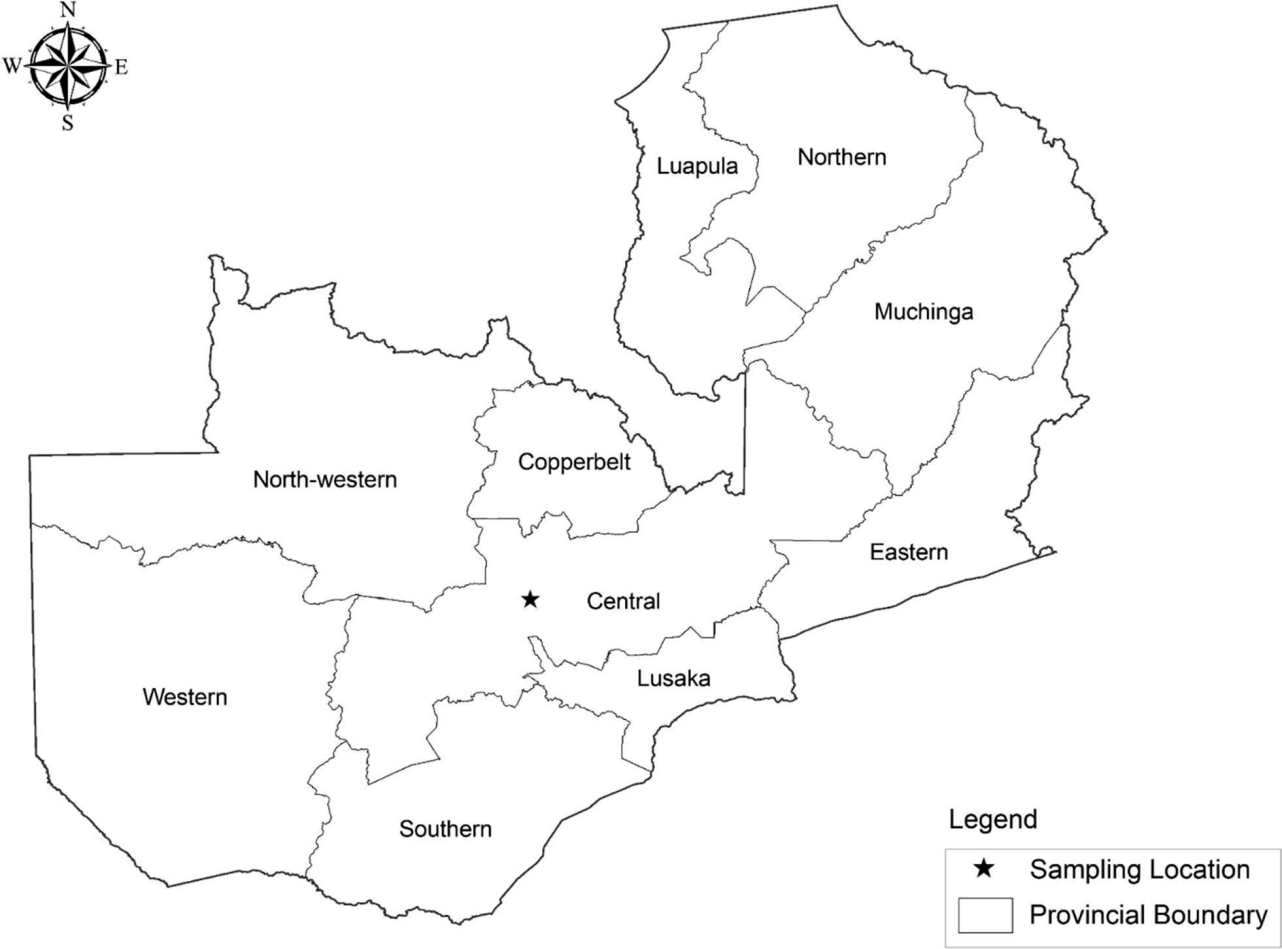
Outbreak and sample collection site

In December 2017, the Central Veterinary Research Institute (CVRI) received samples of skin nodules and scabs from a herd of cattle, initially presumed to have LSD infection. The cattle were a Zebu-Boran crossbreed, of mixed dairy and beef, from Chiyuni veterinary camp, Chief Chitanda area, Mundu, Chibombo District, Central Province of Zambia (Fig. 1).

The affected farm is designated under zone 3 of the East Coast fever (ECF) control strategy in Zambia [13]. This is an epidemic area for ECF, which causes high animal mortalities in Zambia.

The samples initially tested negative by real-time Polymerase Chain Reaction (PCR) for capripoxviruses. The extracted DNA was stored and subsequently analyzed using a recently developed High Resolution Melting (HRM) assay for the simultaneous detection and differentiation of eight poxviruses of medical and veterinary importance [12]. The results prompted a follow-up trip to the farm in April 2018 to clinically examine the animals and obtain additional history and samples.

### Sample collection and DNA extraction

Skin nodules were collected from eight cattle showing recent fresh lesions (Table 1) and were transferred to the CVRI laboratory on ice in a coolbox. The nodules were ground, and 50 mg was homogenized in 2 ml phosphate-buffered saline and centrifuged at 2500 rpm for 10 min at 4 °C. 200 µl of the supernatant was added to 800 µl of lysis buffer RLT Plus (QIAGEN, Germany) and the total nucleic acid extracted using the RNeasy mini kit (QIAGEN, Germany) as previously described [14].

**Table 1:** List of PCPV samples and animals from which specimens were collected for this study in Zambia, 2017–2018

Animal number	Name	Age	Sex	Collection date	Host
1	Lungu	2 years	M	20.12.17	Cattle
2	Mary	2 years 2 mo	F	20.12.17	Cattle
3	Mazete	3 years	F	20.12.17	Cattle
4	Beenzu	5 years	F	24.04.18	Cattle
5	Mary	2 years 6 mo	F	24.04.18	Cattle
6	Mazuba	1 years	M	24.04.18	Cattle
7	Mercy	5 years	F	24.04.18	Cattle
8	Mutinta	2 years	F	24.04.18	Cattle

### Genetic identification using the HRM assay

We tested the extracted DNA using the HRM assay for the simultaneous detection and differentiation of eight poxviruses.[12]. The method can differentially detect members of three different genera of poxviruses; *Capripoxvirus, Orthopoxvirus,* and *Parapoxvirus,* and additionally discriminate the viruses within each of the three genera: cowpox virus (CPXV) and camelpox virus (CMLV) [genus *Orthopoxvirus*]; goatpox virus (GTPV), sheeppox virus (SPPV) and lumpy skin disease virus (LSDV) [genus *Capripoxvirus*]; orf virus (ORFV), pseudocowpox virus (PCPV) and bovine papular stomatitis virus (BPSV) [genus *Parapoxvirus*].

The reaction contained 200 nM of each primer (Table 2), 1 X SsoFast™ EvaGreen® Supermix (Bio-Rad, USA), and 2 µl of the template. Each run included positive control plasmids representing each of the eight pathogens, and a negative control composed of nuclease-free water. Capripoxviruses (GTPV-Denizli, SPPV-Denizli, and LSDV-Ismalia), orthopoxviruses (CMLV- Hadow/01/2012 and CPXV-72/93), and parapoxviruses (ORFV- DZ C-1, PCPV- 2200/12 and BPSV- Stamm M1) were used to produce positive control plasmids [12]. The PCR reactions and melting curve analysis were performed on a real-time PCR machine (CFX96™ Real-Time PCR Detection System, Bio-Rad, USA), following the conditions previously described [12] with slight modifications. Briefly, an initial denaturation step at 95 °C for 4 min was followed by 40 cycles of 95 °C for 1 s, 59 °C for 5 s and 70 °C for 5 s. The PCR products were then denatured at 95 °C for 30 s, cooled down to 65 °C for 60 s, then melted from 65 to 85 °C with an 0.2 °C increments every ten seconds with continuous data acquisition. Data was analyzed using the CFX Manager Software (Bio-Rad, USA), and the Precision Melt Analysis Software (Bio-Rad, USA).

**Table 2:** Primers used in this study for the HRM assay and sequencing

Method	Primers’ ID	5ʹ → 3ʹ sequence	Amplicon size (bp)	Target
HRM	OPV-HRM-For	AGGACTAGCCGCGGTAACTTT	56	Orthopoxviruses
	OPV-HRM-Rev	ACAAGATAGAAGCGATGGATACTT		
	CaPV-HRM-For	TCCTGGCATTTTAAGTAATGGT	100	Capripoxviruses
	CaPV-HRM-Rev	GTCAGATATAAACCCGGCAAGTG		
	PPV-HRM-For	TCGAAGATCTTGTCCAGGAAG	112	Parapoxviruses
	PPV-HRM-Rev	CCGAGAAGATCAACGAGGTC		
Sequencing	ORFV-B2Lf-For	GACCTTCCGCGCTTTAATTT	1210	Parapoxviruses
	ORFV-B2Lf-Rev	CCCGCCTGCTAAAAGACT		

### Sequencing of the B2L gene fragment

The primers used to amplify a fragment of the B2L gene of parapoxviruses [15] are indicated in Table 2. The positive PCR products (1210 base pairs) were purified using the Wizard® SV Gel and PCR Clean-Up System (Promega, USA) and sequenced in both directions by LGC Biosearch Technologies (Germany), using standard Sanger sequencing methods. The sequences were edited and assembled using Vector NTI 11.5 software (Invitrogen). All sequences were submitted to GenBank under accession numbers MT448677 to MT448684.

### Phylogenetic analysis

For comparative analysis, additional partial B2L gene sequences of other parapoxviruses were retrieved from GenBank and screened to remove short and duplicate sequences. The final data set for phylogenetic analyses comprised forty-five sequences, including eight PCPV sequences from this study, seven PCPV of cattle and reindeer, eleven camel contagious ecthyma virus (CCEV), fourteen ORFV, and five BPSV.

Sequences were aligned using the muscle (codon) option in MEGA 7. The aligned sequence file was saved in FASTA format, then converted to a nexus format using Seaview. We then performed the Bayesian phylogenetic inference with BEAST. First, a BEAST file was produced from the nexus file with the BEAUti module using the TN93 + G nucleotide substitution and a UPGMA starting tree. The Markov Chain Monte Carlo method was run with BEAST, for 10,000,000 generations with a sample taken each 10,000 generations. The TRACER program was used to inspect the log files and determine the optimum number of burn-in based on the Effective Sample Sizes (ESS > 200).

TreeAnnotator was used to generate the Maximum Clade Credibility (MCC) after discarding the 2% burn-in. The tree was visualized with the associated meta-data using the ggtree package in R version 3.5.2 [16].

## Results

### Clinical and epidemiological investigations

We found approximately one hundred forty animals at the farm presenting with different stages of infection, from recent to healed lesions, and collected skin nodule samples from eight clinically affected cattle with fresh lesions.

**Fig. 2:**
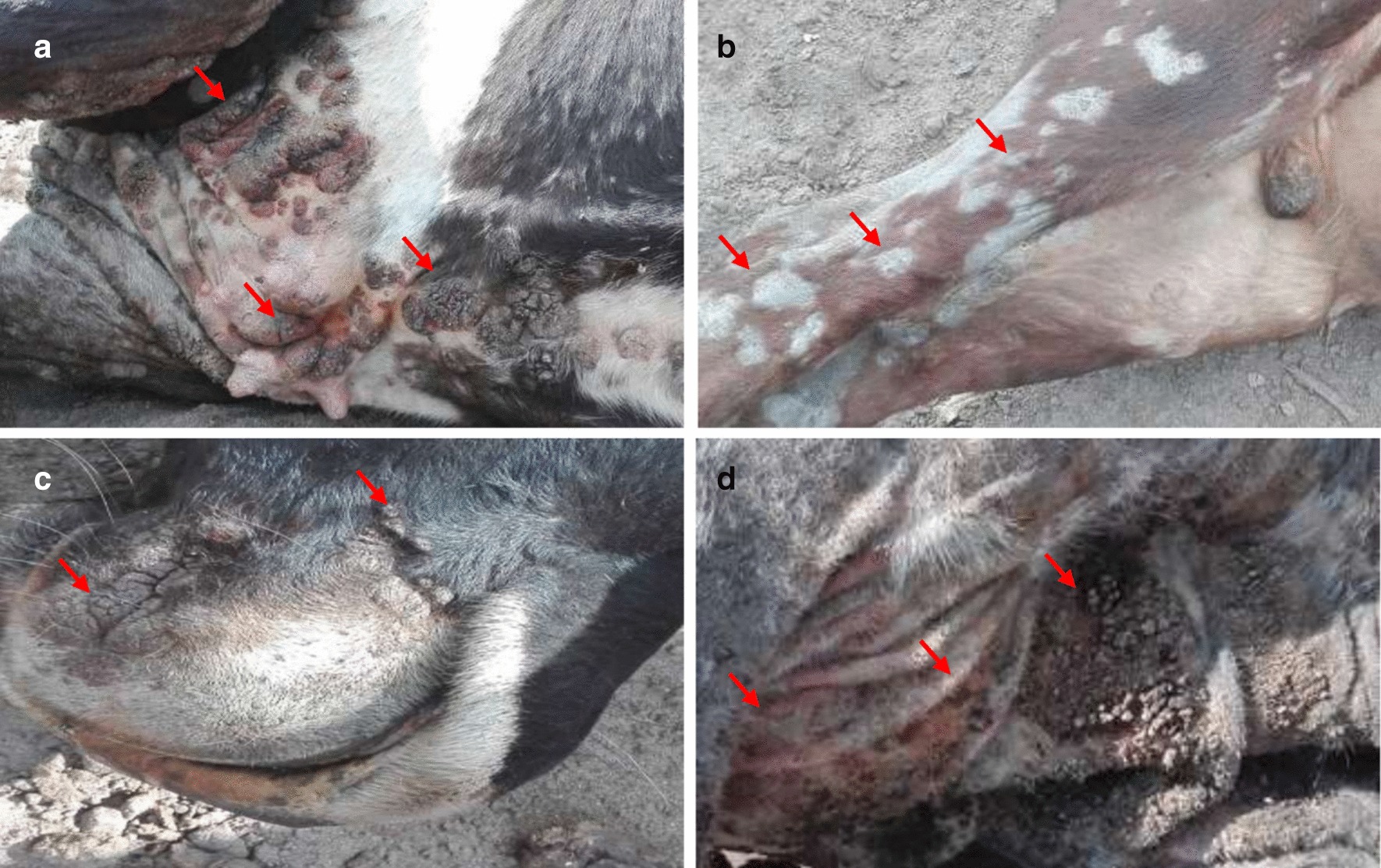
Skin lesions on PCPV infected cattle from Zambia. **a** affected cattle with nodules on the skin and udder. **b** affected calf with scars around the body **c** affected cattle with nodules around the muzzle **d** affected cattle with nodules and dermatitis

The animals had nodular lesions, scabs, and dermatitis on the teats, udder, and other parts of the body, including the sternum, limbs, muzzle, and in skin folds (Fig. 2). Some animals also had enlarged lymph nodes (pre-scapular, parotid, and inguinal). Farmworkers described the presence of itchy nodules on their hands and faces that healed within 1–3 weeks.

The epidemiological history revealed that the farmer had been taking the cattle to graze in the Lukenge swamps since 2011 where they interacted with other neighboring cattle. They first observed skin lesions in 2014 which the farmworkers treated with Oximic plus long-acting (Oxyject 20% plus Sodium diclofenac 0.5%). Since 2014, the farmer reported a loss of approximately 300 animals from a herd of 1900. Because the farm is in an epidemic area for ECF, it was not possible to rule out the involvement of this disease in the high mortalities reported by the farmer. In 2017, as the disease worsened, the farmer finally consulted the veterinary extension services for further investigation.

### Molecular detection

**Fig. 3:**
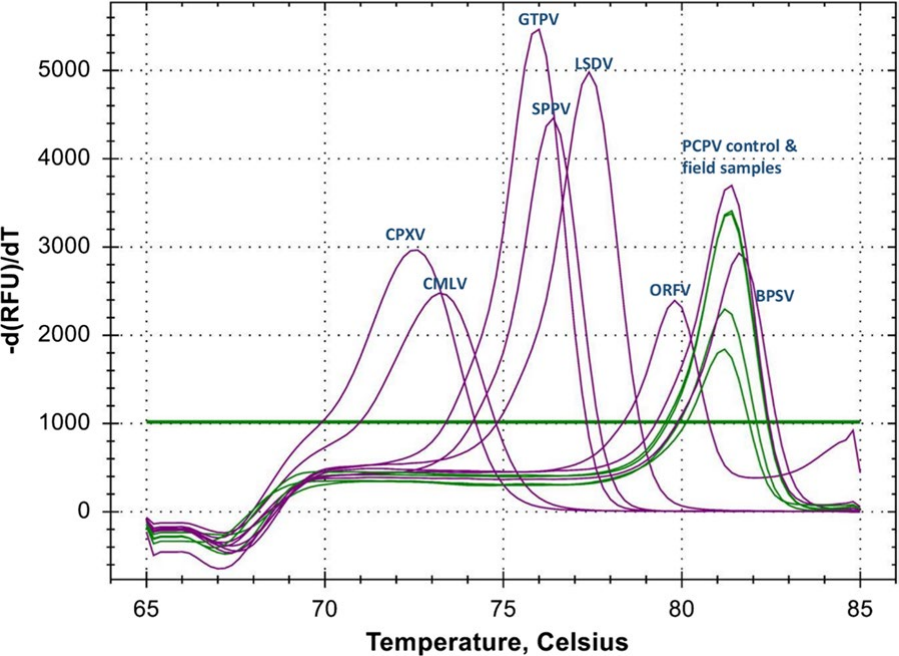
HRM detection of PCPV in selected cattle samples from Zambia. The positive control for each of the eight poxviruses displayed a unique melting peak, shown in purple color. Four samples from Zambia, clustering with PCPV are shown in green color

We detected PCPV DNA in eight out of eight samples using the HRM Assay. Figure 3 shows the amplification curves corresponding to PCPV in all Zambian samples. There was no amplification corresponding to LSDV or Orthopox viruses.

### Molecular characterization and phylogenetic analysis

**Fig. 4:**
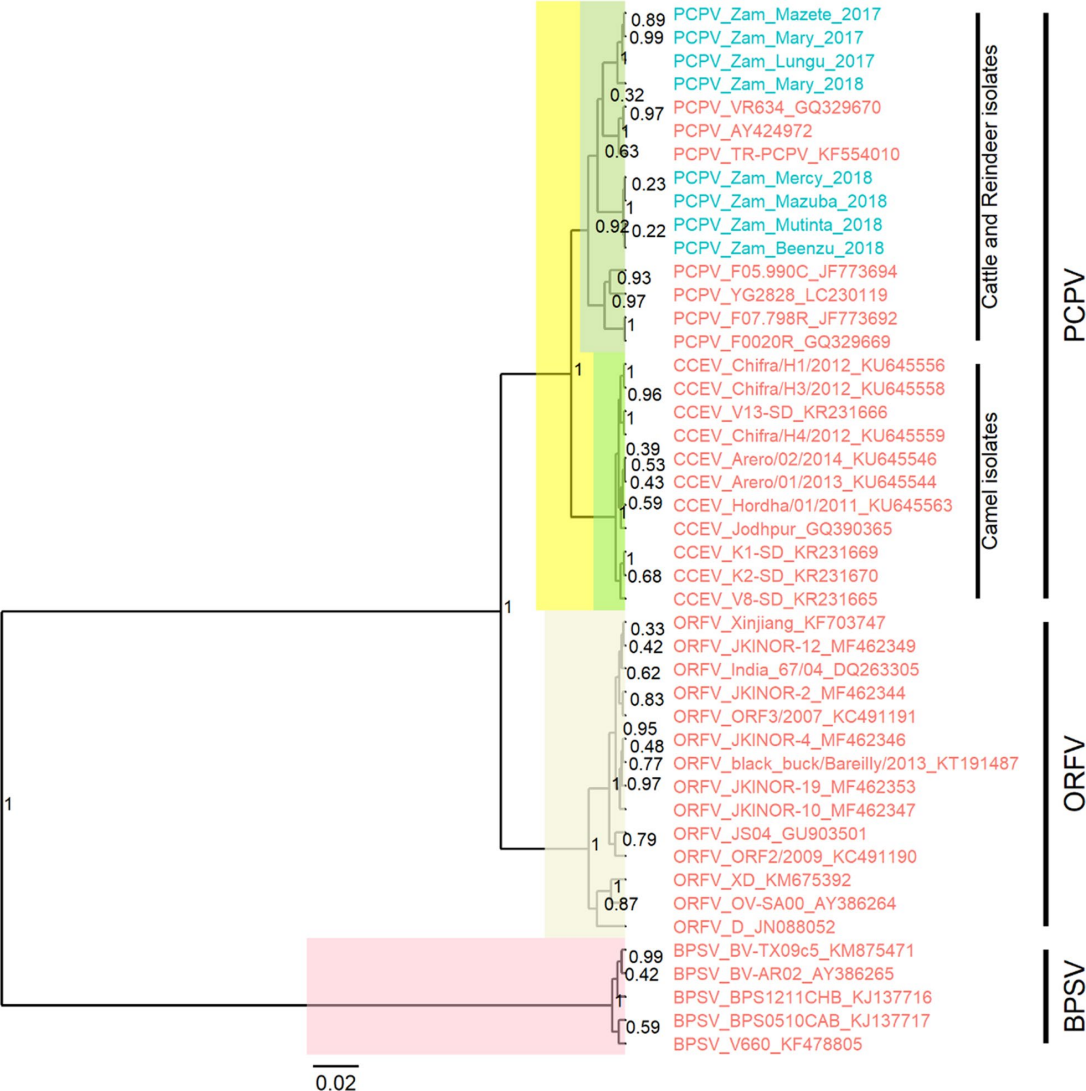
Maximum clade credibility (MCC) tree based on the partial B2L gene sequences of parapoxviruses. The posterior probabilities are plotted as respective nodes labels. The cattle PCPV sequences from Zambia, are highlighted in blue

We successfully amplified and partially sequenced the major envelope protein (B2L gene) in eight out of eight samples. All eight sequences clustered within the PCPV group in the phylogenetic tree (Fig. 4), confirming that the DNA recovered from infected cattle with pox-like lesions belong to PCPV. The tree showed that within the PCPV group, the sequences from camels (CCEV) clustered separately from those collected in cattle and reindeer. All Zambian B2L gene sequences belonged to the group of cattle/reindeer sequences (Fig. 4).

**Fig. 5:**
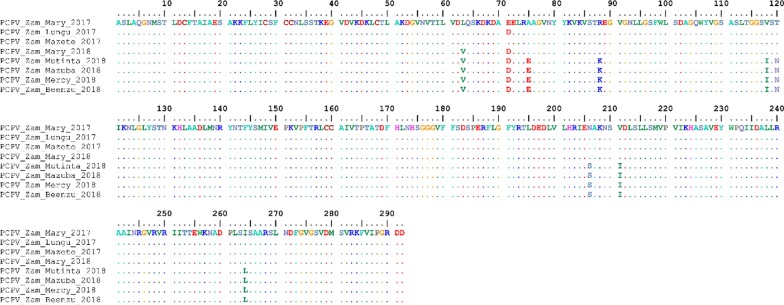
Multiple sequence alignments of the deduced amino acid sequences of the PCPV B2L gene. The PCPV collected in December 2017 and April 2018 in Zambia are compared. Identical amino acids are shown as dots in reference to the first sequence. Note the sequence differences between the 2017and 2018 PCPVs.

Interestingly, sequences from samples collected in December 2017 produced a separate cluster from those collected in April 2018 during the follow-up investigation. The only exception was the sequence of sample Mary 2018, which was closely related to, but different from the 2017 PCPVs (Fig. 4). The sequence alignments of the Zambian PCPVs showed nine polymorphic amino acid sites, with seventeen sites differing at the nucleotide level. Eight of those mutations represented specific changes, with sixteen sites at the nucleotide level, between the 2017 and the 2018 PCPVs. The only exception was the PCPV Mary 2018, which presented only two mutations (four at the nucleotide level) as compared to the 2017 PCPVs (Fig. 5). Mary 2018 and Mary 2017 are from the same animal but collected at two different time points. The mutations seen in the Mary 2018 sequence are among those specific mutations found in the 2018 samples, showing that the PCPV Mary 2018 could be an intermediate variant of the 2017 and 2018 Zambian PCPVs.

## Discussion

In this study, we have detected PCPV DNA in a herd of cattle in Zambia from samples collected based on suspicion of LSD using an HRM assay for differential diagnosis of poxvirus infections. Sequencing and the subsequent phylogenetic analyses confirmed that the DNA in these samples belong to PCPV. In the phylogenetic reconstruction, all Zambian PCPVs clustered with previously published sequences of cattle PCPVs.

Although PCPV in cattle is reportedly present worldwide [17–19], there was no record of the disease in Zambia nor has it been identified in most African countries. Therefore, this marks the first identification of PCPV in the country. The absence of a proper means for differential diagnosis of pox-like diseases in cattle and the lack of confirmed cases for PCPV contributed to the initial misdiagnosis. As LSD is endemic in Zambia, the current cases were mistakenly reported as LSD-suspected, based only on a clinical diagnosis. Similar cases of initial LSD diagnosis have been reported [20]. This underlines the importance of determining the etiology of infections in pox-like skin lesions on cattle.

Our findings highlight the relevance of molecular methods for differential diagnosis and the management of pox diseases in ruminants. This robust HRM assay has enabled differentiation between LSDV and PCPV in a single test and identified PCPV, which otherwise would have gone unnoticed.

In the phylogenetic analyses, PCPVs from samples collected in December 2017 clustered independently from those samples taken from the same farm in April 2018, showing an evolution of the virus during the persistence of the infection on the farm. A close inspection of the sequence alignments showed up to 9 amino acid changes in the partial B2L sequence of PCPVs in samples collected in April 2018 as compared to those collected in December 2017. Interestingly, the analysis of the PCPV B2L gene in samples collected from the same animal at these two-time points showed that two amino acid mutations occurred.

Such genomic changes in the PCPV could suggest either reinfection of the herd or genomic changes of the virus during persistent infections in the herd. A previous report revealed a similar evolution in the ATPase gene of the ORF virus during the persistence of the virus in a sheep herd in Ethiopia [15]. Such alterations in the genome could potentiate the adaptation of the virus to new tissues and promote shedding, thus enhancing its potential spread.

Our field investigations suggested that skin lesions were observed on some cattle in this herd since 2014. However, the farmer consulted local veterinary services only when the health conditions of cattle had worsened. The affected cattle had lesions that were not only confined to the teats and udder, but, were present on other parts of the animal, including the limbs, mouthparts, ventrum, and skin folds, suggesting a severe form of the disease. PCPV has previously been isolated from atypical sites apart from the teats and udder [18, 19, 21]. As PCPV is a zoonotic disease, humans coming into direct contact with infected animals are at risk of contracting the infection [22]. We could not find an active case of transmission to humans, and therefore, no samples were collected.

Nevertheless, it came to our attention that the farmworkers previously had sores mainly on their hands and forearms, and sometimes the face. Those lesions cleared within a few weeks without treatment. This observation is consistent with previous reports showing that PCPV lesions in humans usually resolve quickly [23]. It is advisable to handle infected animals with caution to reduce the risks.

## Conclusion

This first detection and characterization of PCPV demonstrates the power of molecular methods for the differential diagnosis of pox diseases in cattle. The correct identification of the causative agent of pox-like lesions in cattle is essential to allow for proper veterinary intervention, including vaccination of non-infected surrounding herds in the case of LSD. Similarly, strict hygiene measures are needed to prevent transmission to humans when PCPV infection is detected. Country-wide surveillance may be crucial to investigate the prevalence of PCPV infections among the cattle population and identify infection risks for other animals and humans in Zambia.

## Data Availability

DNA sequences generated and analyzed under the current study are available in GenBank under accession numbers MT448677 to MT448684.

## Abbreviations

BoHV: Bovine herpes virus
BPSV: Bovine papular stomatitis virus
CCEV: Camel contagious ecthyma virus
CMLV: Camelpox virus
CPXV: Cowpox virus
CVRI: Central Veterinary Research Institute
DNA: Deoxyribonucleic acid
ECF: East coast fever
ESS: Effective sample sizes
GTPV: Goatpox virus
HRM: High-resolution melting
LSDV: Lumpy skin disease virus
MCC: Maximum clade credibility
ORFV: Orf virus
PCR: Polymerase chain reaction
PCPV: Pseudocowpox virus
PPV: Parapoxvirus
PVNZ: Parapoxvirus of red deer in Newzealand
SPPV: Sheeppox virus
